# Preoperative prognostic nutritional index predicts short-term complications after radical resection of distal cholangiocarcinoma

**DOI:** 10.3389/fsurg.2022.1091534

**Published:** 2023-01-10

**Authors:** Yulong He, Haoran Liu, Yuhu Ma, Jianlong Li, Jinduo Zhang, Yanxian Ren, Chunlu Dong, Bing Bai, Yong Zhang, Yanyan Lin, Ping Yue, Wenbo Meng

**Affiliations:** ^1^ The First Clinical Medical College, Lanzhou University, Lanzhou, China; ^2^ Department of General Surgery, The First Hospital of Lanzhou University, Lanzhou, China; ^3^ Gansu Province Key Laboratory of Biological Therapy and Regenerative Medicine Transformation, Lanzhou, China

**Keywords:** distal cholangiocarcinoma, postoperative complication, prognostic nutritional index, total bilirubin, decision tree

## Abstract

**Background:**

The occurrence of postoperative complications of distal cholangiocarcinoma (dCCA) is an indicator of poor patient prognosis. This study aimed to determine the immune-nutritional indexes (INIs) that can predict short-term postoperative complications.

**Methods:**

A retrospective analysis of 148 patients with dCCA who were operated radical pancreaticoduodenectomy at the First Hospital of Lanzhou University from December 2015 to March 2022 was conducted to assess the predictive value of preoperative INIs and preoperative laboratory tests for short-term postoperative complications, and a decision tree model was developed using classification and regression tree (CART) analysis to identify subgroups at risk for overall complications.

**Results:**

In this study, 83 patients (56.08%) experienced overall complications. Clavien-Dindo grade III-V complications occurred in 20 patients (13.51%), and 2 patients died. The areas under curves (AUCs) of the preoperative prognostic nutritional index (PNI), controlling nutritional status (CONUT) score, and neutrophil-to-lymphocyte ratio (NLR) were compared; the PNI provided the maximum discrimination for complications (AUC = 0.685, 95% CI = 0.600–0.770), with an optimal cutoff value of 46.9, and the PNI ≤ 46.9 group had higher incidences of overall complications (70.67% vs. 40.00%, *P* < 0.001) and infectious complications (28.77% vs. 13.33%, *P* = 0.035). Multivariate logistic regression analysis identified PNI (OR = 0.87, 95% CI: 0.80–0.94) and total bilirubin (OR = 1.01, 95% CI: 1.00–1.01) were independent risk factors for overall complications (*P* < 0.05). According to CART analysis, PNI was the most important parameter, followed by the total bilirubin (TBIL) level. Patients with a PNI lower than the critical value and TBIL higher than the critical value had the highest overall complication rate (90.24%); the risk prediction model had an AUC of 0.714 (95% CI, 0.640–0.789) and could be used to stratify the risk of overall complications and predict grade I-II complications (*P* < 0.05).

**Conclusion:**

The preoperative PNI is a good predictor for short-term complications after the radical resection of dCCA. The decision tree model makes PNI and TBIL easier to use in clinical practice.

## Introduction

Cholangiocarcinoma (CCA) is a highly lethal malignancy that may occur anywhere within the biliary tree and/or liver parenchyma (1). The incidence of CCA is gradually increasing worldwide (2). Depending on the anatomical site of origin, CCA is classified as intrahepatic, perihilar, or distal cholangiocarcinoma (dCCA), and dCCA accounts for approximately 20% of CCA cases (3). Due to the location and invasive nature of dCCA, such patients often present with locally advanced or metastatic disease. Surgery through pancreaticoduodenectomy is the only curative option (4). Postoperative complications are one of the factors for poor prognosis in many cancers, and the occurrence of postoperative complications will prolong the postoperative recovery time, increase the economic burden, and result in poor long-term prognosis (5). It has been reported that postoperative complications may lead to systemic inflammation, which may reduce the immune response to cancer (6).

Patients with cancer often suffer from severe nutritional deficiencies, malnutrition, and low immunity will adversely affect the development of cancer patients, increase the incidence of infection, length of hospital stay, and risk of death (7), and may lead to higher rates of complications (8). In addition, We reviewed the relationship between preoperative immuno-nutritional related indicators and the occurrence of complications and prognosis in patients with CCA, where the association of prognostic nutritional index (PNI), nutritional control status (CONUT) score, neutrophil-to-lymphocyte ratio (NLR)and dCCA needs to be further investigated. This research aimed to evaluate the ability of preoperative immune-nutritional indexes (INIs) to predict postoperative complications after radical resection of dCCA and establish a decision tree model subgroups according to risk level to provide a basis for the prevention and treatment of postoperative complications, guide health education and prognosis management for patients, and accelerate patient recovery.

## Methods

This retrospective study was conducted in The First Hospital of Lanzhou University in China, the study followed the Declaration of Helsinki and was approved by the Ethics Committee of The First Hospital of Lanzhou University (LDYY2022-412).

### Patient enrollment

**Figure 1:**
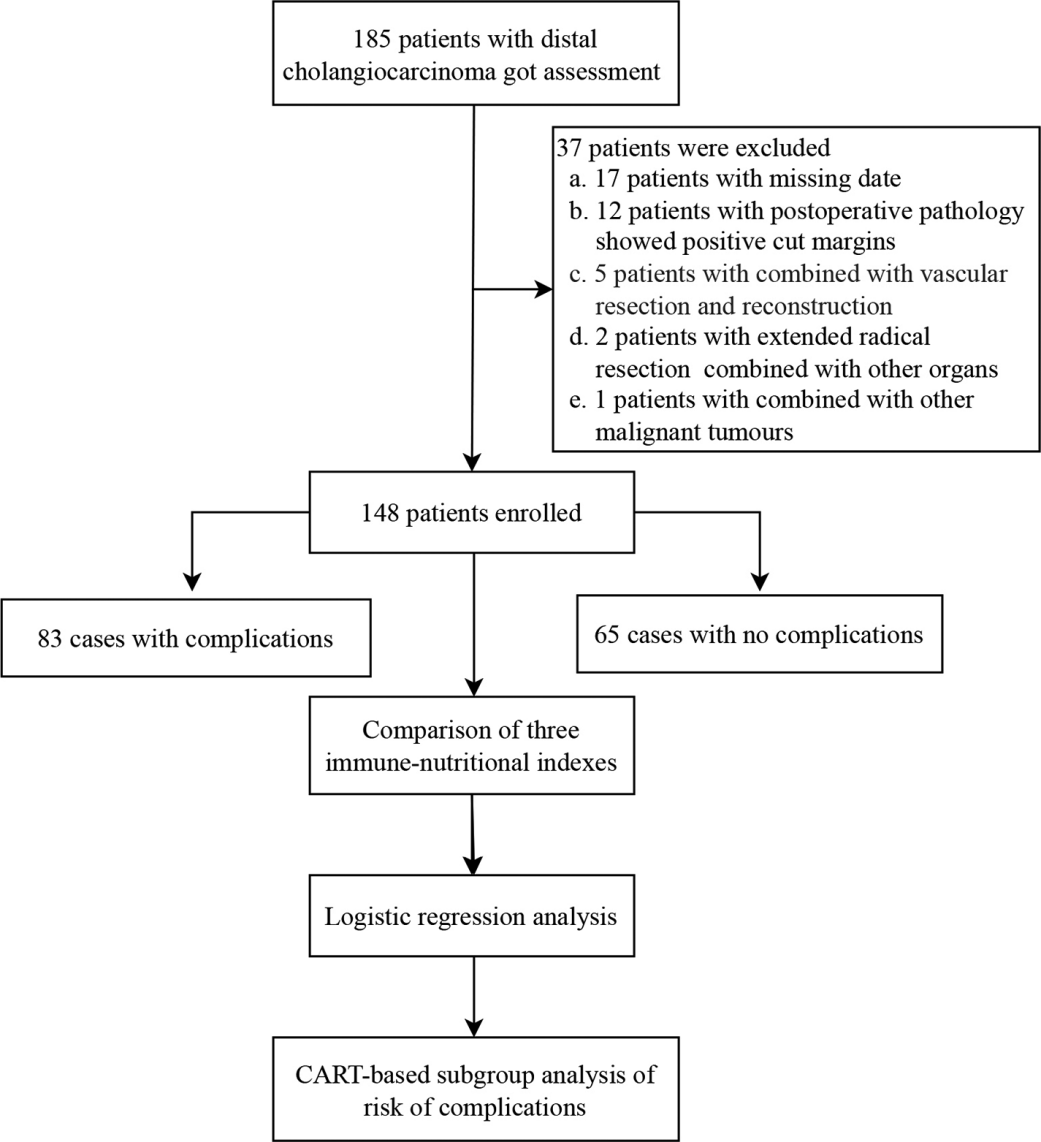
Patient enrollment flowchart.

This retrospective study included patients with pancreaticoduodenectomy for dCCA at the First Hospital of Lanzhou University from December 2015 to March 2022. Patients who met the following criteria were included: (1) postoperative pathological examination consistent with a diagnosis of cholangiocarcinoma; (2) no distant metastasis found during the operation; (3) no severe heart, lung, kidney, or brain dysfunction before the operation (without combined organ dysfunction such as heart failure, respiratory failure, renal failure, disorientation and stress disorder, etc.); and (4) complete clinical medical records. Subjects with the following were excluded: (1) combined vascular resection and reconstruction; (2) extended radical resection combined with resection of other organs; (3) postoperative pathology showing a positive cut margin; (4) previously complicated with other malignant tumors; and (5) lack of clinical medical records. Finally, 148 patients were enrolled in this study (Figure 1).

### Data collection

Hospital records were retrospectively evaluated for baseline information, laboratory tests, postoperative pathological factors, and postoperative complications, as well as PNI, CONUT score, and NLR when the patient was first admitted to the hospital. PNI was calculated using the following formula: serum albumin (g/L) + 5 × total lymphocyte count (10^9 ^L^−1^) (9). The CONUT score consists of three parameters: serum albumin, total peripheral lymphocyte count, and total cholesterol. Postoperative complications were defined as those occurring within 2 weeks after surgery and were assessed by the Clavien‒Dindo classification (10). Abdominal infection was defined by a positive abdominal culture collection, persistent fever or elevated leucocyte count, and effective antibiotic treatment, with or without bacteremia or a liver abscess, together with the exclusion of other infections including pulmonary and wound infections (11). Postoperatively, delayed gastric emptying was defined as the need for gastric tube placement for more than 3 days after the operation, the need for repeat catheter placement due to vomiting and other reasons after extubation, and an inability to consume solids for 7 days or longer after the operation (12).

## Statistical analysis

All statistical analyses in this study were conducted using R (version 4.1.3). Quantitative data are presented as median (interquartile range) and were compared using the Wilcoxon rank sum test. Qualitative data were compared by the chi-square test and are expressed as frequency and percentage (%). *P* values < 0.05 were considered statistically significant. The potential predictive value of preoperative INIs for overall complications was assessed by the area under the receiver operating characteristic (ROC) curve. The Youden index was used to select the optimal cutoff value, which was set as the value with the maximum summed sensitivity and specificity value. Logistic regression analysis was used to identify risk factors for overall complications. *P* values < 0.05 were considered statistically significant, and independent risk factors for overall complications after surgery for dCCA were identified by univariate and multifactorial analysis.

The classification and regression tree (CART) model was constructed using the R package “party” (version 4.1.3) by identifying independent risk factors for overall complications through logistic regression analysis. CART, a machine learning method for constructing predictive models that simulate clinical decision-making processes (13), is easy to understand and implement. The decision tree construction process is recursive. Thus, the parameter most closely related to the result is extracted as the first node, When there is no correlation between the predictor variable and the outcome variable, the algorithm stops, and the *p* values show the relationship between the preoperative variables and the postoperative complications. Finally, the AUC was used to evaluate the accuracy of the decision tree in predicting overall complication outcomes.

## Results

### Patient characteristics

This study included 148 patients, 88 males, and 60 females, the age range of 28–78 years. Clinical characteristics, such as preoperative blood routine, biochemical tests, coagulation indicators, tumor markers, and postoperative pathology, were collected, and the clinical characteristics of the patients were summarized and analyzed (Table 1).

**Table 1:** Clinical characteristics of the patients at baseline.

Characteristics	Complication (*n* = 83)	*N*-Complication (*n* = 65)	*P* Value
Age (years), median (IQR)	63 (57.5–61)	61 (54–69)	0.234
Male sex, *n* (%)	53 (63.86%)	35 (53.85%)	0.288
Laboratory examination, median (IQR)			
Erythrocyte (109/L)	4.31 (4.04–4.71)	4.41 (4.02–4.86)	0.354
Leukocyte (109/L)	6.35 (5.08–9)	5.30 (4.53–6.64)	0.002
Hemoglobin (g/L)	137 (131–148.5)	139 (126–154)	0.857
Albumin (g/L)	39.7 (36.8–42.9)	41 (39–44)	0.006
Total lymphocyte (109/L)	1.27 (0.97–1.41)	1.4 (1.22–1.7)	0.006
Aspartate transaminase (U/L)	129 (77–195.5)	114 (74–251)	0.845
Total bilirubin (µmol/L)	267.9 (162.35–368.1)	154.8 (90–219)	<0.001
Cholesterol (mmol/L)	4.77 (4.12–6.47)	4.87 (3.94–6.15)	0.759
Triglycerides (mmol/L)	1.71 (1.12–2.47)	2.11 (1.56–2.94)	0.030
Prothrombin time (S)	11.4 (10.8–12.8)	11.2 (10.7–12.1)	0.050
Carbohydrate antigen 199 (U/ml)	129 (35.75–272.55)	86.9 (56.5–190)	0.655
Comorbidities, *n* (%)			
Diabetes	6 (7.23%)	6 (7.23%)	0.889
Hypertension	18 (21.69%)	16 (24.62%)	0.823
Bile duct stones	8 (9.64%)	3 (4.62%)	0.401
Cholecystolithiasis	10 (12.05%)	7 (10.78%)	1.000
Hepatitis	4 (4.82%)	3 (4.62%)	1.000
Pathological factors, *n* (%)			
TNM staging			0.905
0-IIb	62 (74.70%)	50 (76.92%)	
IIb-IV	21 (25.30%)	15 (23.08%)	
Vascular invasion	17 (20.48%)	10 (15.38%)	0.589
Lymph node metastasis	28 (33.73%)	18 (27.69%)	0.542
Differentiation			0.008
Well	7 (8.43%)	5 (7.69%)	
Moderate	23 (27.72%)	14 (21.54%)	
Low-middle	48 (57.83%)	29 (44.62%)	
Poorly	5 (6.02%)	17 (26.98%)	

Data are presented as *n* (%) or median (IQR). IQR, Interquartile range.

In this study, 83 patients (56.08%) had overall complications, of which 20 patients (13.51%) had Clavien‒Dindo grade III-V complications, and 2 patients died. There were 60 cases (40.54%) of pancreatic leakage, 35 cases (23.65%) of biochemical leakage, 24 cases (16.22%) of Grade B pancreatic leakage, 1 case (0.68%) of Grade C pancreatic leakage, 33 cases (22.30%) of delayed gastric emptying, 23 cases (15.54%) of abdominal infection, and 18 cases (12.26%) of abdominal bleeding. There were 13 cases (8.78%) of bile leakage, 11 cases (7.43%) of pulmonary infection, and 4 cases (2.70%) of wound infection (Table 2).

**Table 2:** Postoperative complications.

	All (*n* = 148)	CD Grades I-II	CD Grades III-V
Postoperative complication	83 (56.08%)	63 (42.57%)	20 (13.51%)
Intraperitoneal hemorrhage	18 (12.16%)	4 (2.70%)	14 (9.46%)
Pancreatic leakage	60 (40.54%)	46 (31.08%)	14 (9.46%)
Biochemical leakage	35 (23.65%)	31 (20.95%)	4 (2.70%)
Grade B	24 (16.22%)	15 (10.14%)	9 (6.08%)
Grade C	1 (0.68%)	0	1 (0.68%)
Bile leakage	13 (8.78%)	8 (5.41%)	5 (3.37%)
Abdominal infection	23 (15.54%)	12 (8.11%)	11 (7.43%)
Wound infection	4 (2.70%)	2 (1.35%)	2 (1.35%)
Pulmonary infection	11 (7.43%)	4 (2.70%)	7 (4.73%)
Delayed gastric emptying	33 (22.30%)	25 (16.89%)	8 (5.41%)

Data are presented as *n* (%). CD, Clavien–Dindo classification.

### Predictive values of INIs for postoperative complications

**Figure 2:**
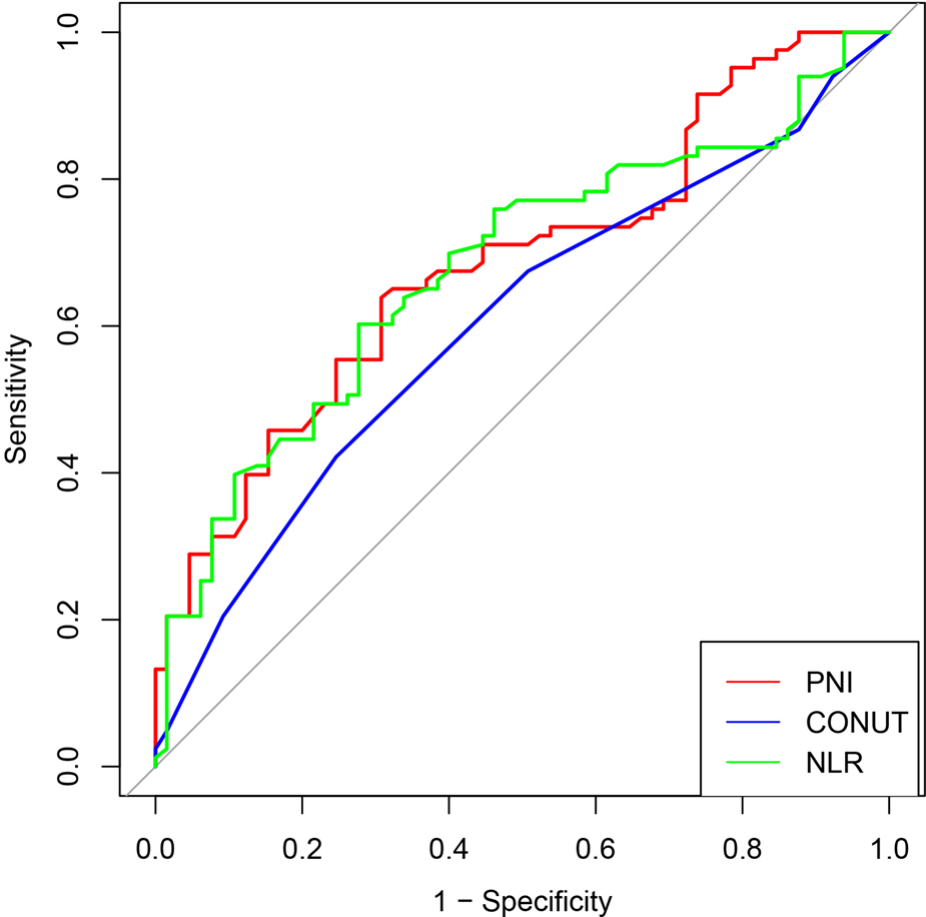
ROC curves for INIs related to postoperative complications.

For investigating the predictive value of preoperative INIs for short-term complications after radical surgery for dCCA and evaluating the correlation between the predictor and outcome variables, we plotted ROC curves for PNI, CONUT, and NLR. The results show that PNI has the highest differentiation (AUC = 0.685, 95% CI = 0.600–0.770), followed by NLR (AUC = 0.678, 95% CI = 0.592–0.765) and CONUT (AUC = 0.603, 95% CI = 0.514–0.692). A comparison of the AUC of each curve showed that PNI correlated more strongly with complications than CONUT and NLR. PNI has a better predictive value for postoperative complications, with an optimal cutoff value of 46.9, corresponding to the maximum Youden index (Figure 2).

### Correlations between PNI and clinical characteristics

**Figure 3:**
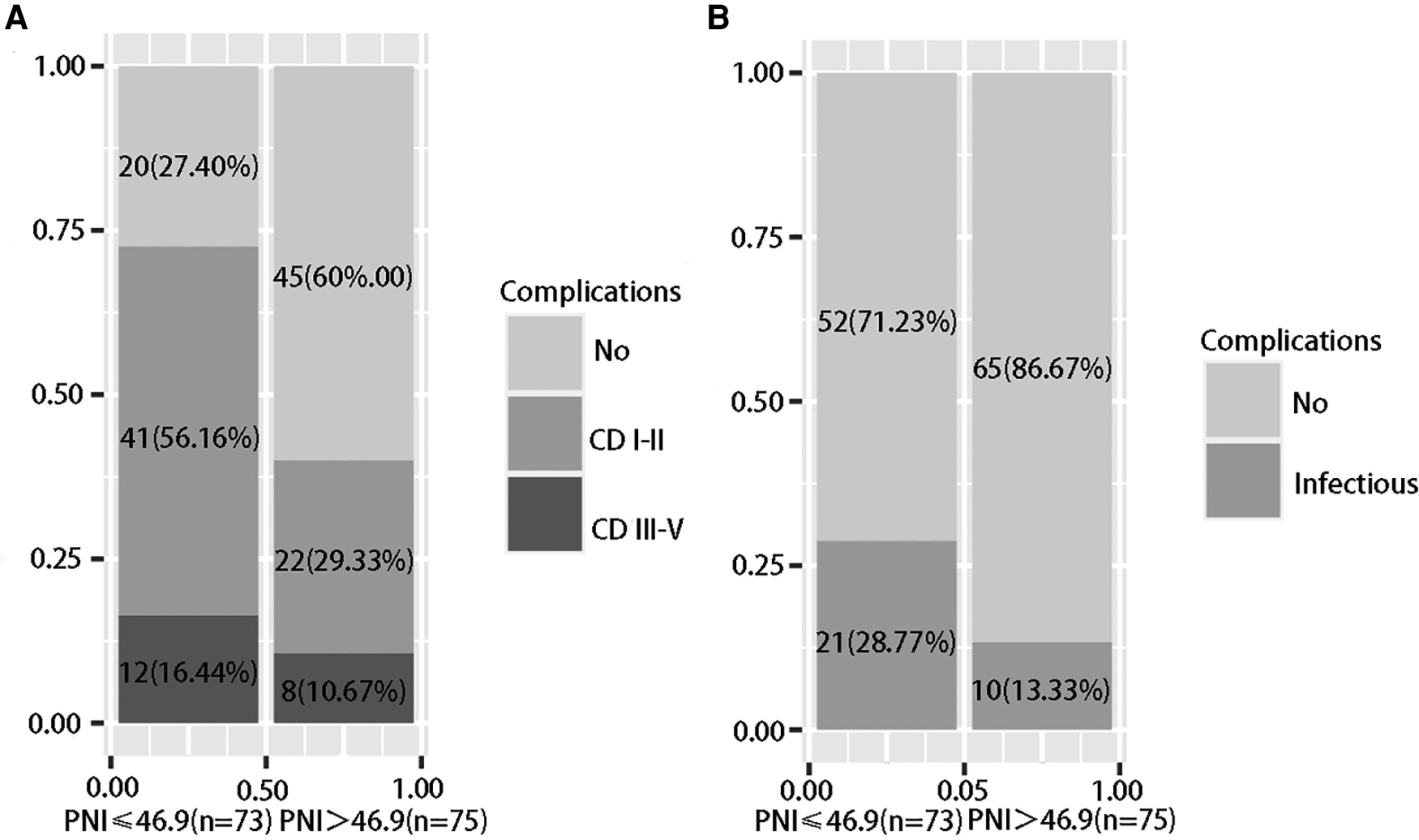
(**A**) Frequency of postoperative complications; (**B**) Frequency of infectious complications.

The patients were divided into a low PNI group (PNI ≤ 46.9, *n* = 73) and a high PNI group (PNI > 46.9, *n* = 75) based on the optimal cutoff value. The low PNI group had lower erythrocyte counts, higher leukocyte counts, lower hemoglobin levels, lower albumin levels, lower total lymphocyte counts, higher total bilirubin levels, and longer prothrombin times. There were no significant differences in pathological characteristics between the two groups. In terms of postoperative complications, the low PNI group had a higher occurrence of complications (70.67%, *P* < 0.001), and a low PNI had a stronger ability to predict overall complications [Figure 3(a)], and the low PNI group had a higher occurrence of infectious complications (28.77%, *P* = 0.035) [Figure 3(b)]. A low PNI had predictive value for overall complications and infectious complications (Table 3).

**Table 3:** Clinicopathological characteristics of the low PNI group and the high PNI group.

Characteristics	PNI ≤ 46.9 (*n* = 73)	PNI > 46.9 (*n* = 75)	*P* Value
Laboratory examination, median (IQR)			
Erythrocyte (10^9^/L)	4.22 (3.93–4.55)	4.6 (4.09–5.02)	0.015
Leukocyte (10^9^/L)	6.35 (5.1–9.13)	5.44 (4.75–6.99)	0.023
Hemoglobin (g/L)	137 (122–145)	141 (130–145)	0.022
Albumin (g/L)	38.8 (35.9–39.9)	43.3 (41.05–45.3)	<0.001
Total lymphocyte (10^9^/L)	1.27 (0.87–1.37)	1.42 (1.22–1.87)	<0.001
Aspartate transaminase (U/L)	146 (75–260)	118 (74–198.5)	0.444
Total bilirubin (µmol/L)	223.2 (137.5–380.5)	194.1 (124.65–262.9)	0.049
Cholesterol (mmol/L)	4.87 (4–6.15)	4.79 (4.03–6.49)	0.766
Triglycerides (mmol/L)	1.72 (1.34–2.76)	2.04 (1.27–2.85)	0.914
Prothrombin time (S)	11.5 (11–12.9)	11 (10.55–12.15)	0.002
Carbohydrate antigen 199 (U/ml)	136.1 (50.3–283.4)	82.1 (43.05–183.45)	0.163
Pathological factors, *n* (%)			
TNM staging			0.922
0-IIb	56 (76.71%)	56 (74.67%)	
IIb-IV	17 (23.29%)	19 (25.33%)	
Vascular invasion	10 (13.70%)	17 (22.67%)	0.230
Lymph node metastasis	17 (23.29%)	29 (38.67%)	0.065
Differentiation			0.889
Well	7 (9.59%)	5 (6.67%)	
Moderate	19 (26.03%)	18 (24.00%)	
Low-middle	37 (50.68%)	40 (53.33%)	
Poorly	10 (13.70%)	12 (16.00%)	
Complications, *n* (%)			
All Complication	53 (70.67%)	30 (40.00%)	<0.001
Clavien-Dindo			<0.001
No	20 (27.40%)	45 (60.00%)	
Grade I-II	41 (56.16%)	22 (29.33%)	
Grade III-V	12 (16.44%)	8 (10.67%)	
Serious complications	12 (16.44%)	8 (10.67%)	0.432
Infectious Complication	21 (28.77%)	10 (13.33%)	0.035

Data are presented as *n* (%) or median (IQR). IQR, Interquartile range.

### Logistic regression analysis to identify risk factors for overall complications

This study used logistic regression analysis to identify risk factors for postoperative complications (Table 4). Univariate analysis showed that prognostic nutritional index (OR = 0.85, 95% CI: 0.79–0.93), total bilirubin (OR = 1.00, 95% CI: 1–1.01), leukocyte count (OR = 1.28, 95% CI: 1.09–1.49), prothrombin time (OR = 1.34, 95% CI: 1.06–1.70) and triglycerides (OR = 1.51, 95% CI: 1.04–2.19) were influencing factors for overall complications (*P* < 0.05). The above influencing factors were included in multivariate logistic regression analysis, and the results showed that prognostic nutritional index (OR = 0.87, 95% CI: 0.80–0.94) and total bilirubin (OR = 1.01, 95% CI: 1.00–1.01) were independent risk factors for overall complications (*P* < 0.05).

**Table 4:** Logistic regression analysis to identify risk factors for overall complications.

Characteristics	Univariate analysis			Multivariate analysis		
	OR	95%CI	*P* Value	OR	95%CI	*P* Value
Age (years)	1.02	0.99–1.06	0.239			
Sex	0.66	0.34–1.28	0.219			
Prognostic nutritional index	0.85	0.79–0.93	<0.001	0.87	0.80–0.94	0.001
Leukocyte (10^9^/L)	1.28	1.09–1.49	0.002	1.19	1.01–1.44	0.052
Erythrocyte (10^9^/L)	0.70	0.39–1.27	0.245			
Hemoglobin (g/L)	1.00	0.98–1.01	0.658			
Aspartate transaminase (U/L)	1.00	0.99–1.01	0.992			
Alanine transaminase (U/L)	1.00	0.99–1.01	0.389			
Total bilirubin (µmol/L)	1.00	1–1.01	0.001	1.01	1.00–1.01	0.017
Total protein (g/L)	0.99	0.94–1.03	0.553			
Cholesterol (mmol/L)	1.00	0.88–1.14	0.993			
Uric acid (µmol/L)	1.00	1–1.01	0.540			
Glucose (mm/L)	1.01	0.88–1.15	0.896			
Triglycerides (mm/L)	1.51	1.04–2.19	0.028	1.24	0.79–1.94	0.349
Prothrombin time (S)	1.34	1.06–1.70	0.014	1.10	0.85–1.50	0.478
International normalized ratio	1.59	0.49–5.19	0.441			
Carbohydrate antigen 199 (U/ml)	1.00	0.99–1.01	0.792			
Carcinoembryonic antigen (ng/MI)	0.98	0.94–1.03	0.521			

### CART-based subgroup analysis of overall complication risk

**Figure 4:**
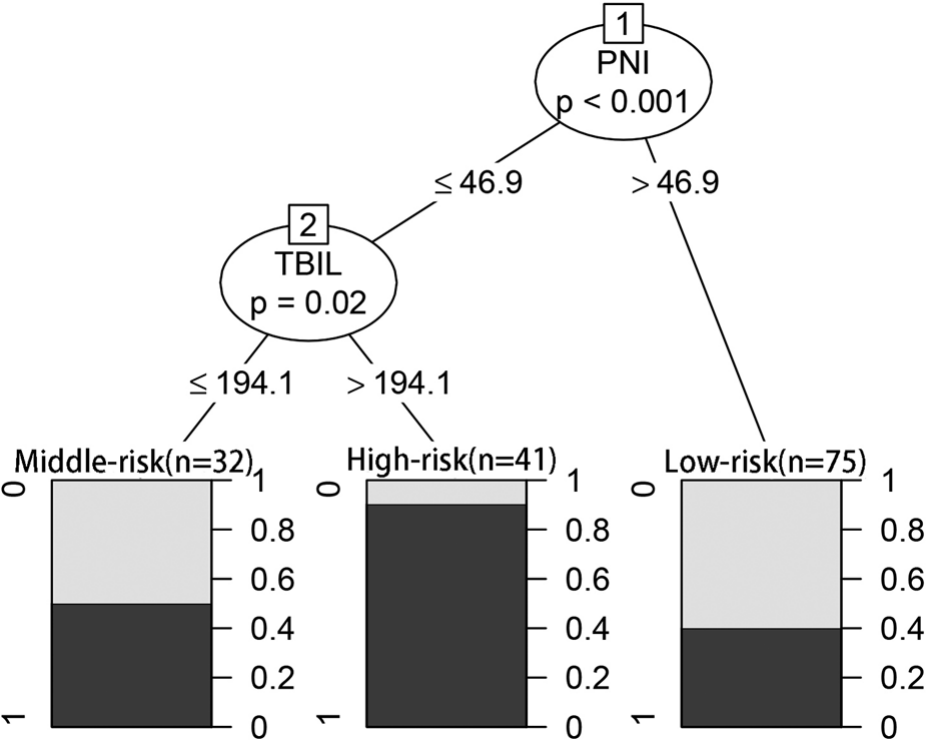
Prediction model for overall complication using CART analysis.

**Figure 5:**
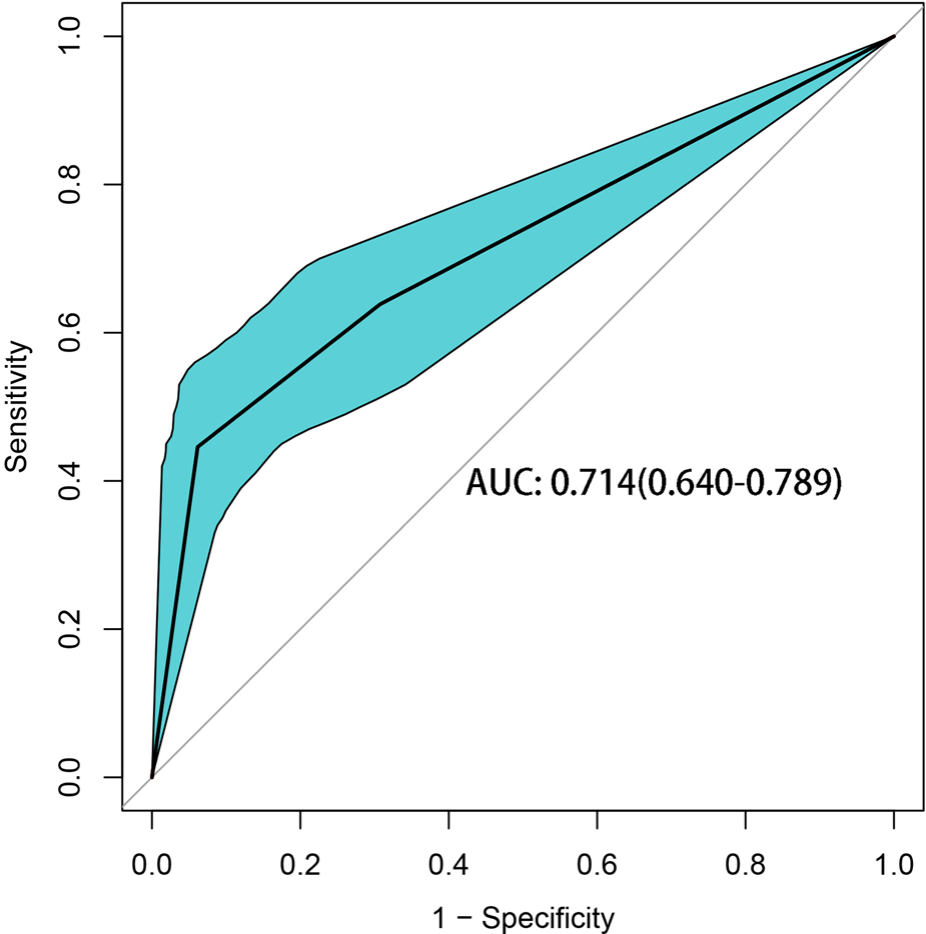
ROC curve of the decision tree.

In the CART algorithm, we used the important variables PNI and total bilirubin (TBIL). The PNI was the most critical parameter for overall complications, and the optimal cutoff value was 46.9. Based on the correlation between the predictor and outcome variables, the CART algorithm selected the optimal cut-off point for TBIL levels for all patients, with an optimal cut-off value of 194.1 µmol/L (Figure 4). Therefore, patients with a PNI lower than the cutoff value and TBIL higher than the cutoff value had the highest complication rate (high-risk group: 37/41 patients, 90.24%), followed by the medium-risk group and low-risk group having overall complication rates of 50% (16/32 patients) and 40% (30/75 patients), respectively. The risk prediction model had an AUC of 0.714 (95% CI, 0.640–0.789) and had a good effect in predicting the overall risk of complications (Figure 5). We analyzed the relationship between risk groups and Clavien‒Dindo grades established by the decision tree model (Table 5), which could stratify overall complication risk groups and predict grade I-II complications (*P* < 0.05).

**Table 5:** Relationship between risk group established by the prediction model and complications.

Complications	High-risk group (*n* = 41)	Middle-risk group (*n* = 32)	Low-risk group (*n* = 75)	*P* value
All complications				<0.001
Yes (*n* = 83)	37	16	30	
No (*n* = 65)	4	16	45	
CD Grades I-II				<0.001
Yes (*n* = 63)	28	13	22	
No (*n* = 85)	13	19	53	
CD Grades III-V
Yes (*n* = 20)	9	3	8	0.175
No (*n* = 128)	32	29	67	

Data are presented as *n* (%). CD, Clavien–Dindo classification.

## Discussion

The prognosis of dCCA is poor, and the median survival time of patients with the unresectable disease is less than one year (14). Patients with dCCA who underwent radical pancreaticoduodenectomy, especially R0 resection, can achieve long-term survival (15). However, postoperative complications may lead to the deterioration of long-term survival, more use of hospital resources, and an increase in the postoperative readmission rate (16). Effective preoperative differentiation and intervention of high-risk patients can be of great benefit to both clinicians and patients, and this study focuses on identifying preoperative PNI and TBIL levels as valid indicators for predicting short-term complications after radical surgery for dCCA.

The PNI was first proposed in 1980 by Onodera et al. (17) for preoperative nutritional status assessments and surgical risk predictions in gastrointestinal surgery. Serum albumin has been recognized as a prognostic indicator of various diseases and has been widely used to assess nutritional status and prognosis (18,19). Lymphocytes play an important role in tumor-related immunology and have strong antitumor immune functions (20). Therefore, PNI reflects the nutritional and immune status of patients and has been widely used as a prognostic indicator for various cancer patients (21–24). Some previous studies have found that a low PNI is positively correlated with poor prognosis in patients with biliary tract cancer, and the use of this parameter has shown the potential to improve prediction and identify high-risk patients more accurately and precisely (25). A meta-analysis found that PNI was an independent prognostic factor for overall survival and postoperative complications in cancer patients. In addition, subgroups with cutoff values less than 45 reported higher OR values, which indirectly confirmed that a low PNI was indeed associated with poor prognosis in some cancers (26).

However, this value has not been well-studied in patients with dCCA. We compared the correlation and discrimination between the INIs and complications and selected the PNI to distinguish between malnourished and well-nourished patients. Malnutrition is associated with an increased risk of perioperative morbidity and mortality (27) and is a risk factor for postoperative complications (28). A valid and objective preoperative nutritional risk assessment is of great benefit to both clinicians and patients, helping clinical doctors establish the corresponding diagnosis and treatment strategy to achieve improved clinical outcomes. The mechanism by which PNI affects complications after radical pancreaticoduodenectomy for dCCA is not very clear and may be related to albumin and lymphocyte levels, thus reflecting the nutritional and immune status of patients.

The TBIL level can be used to assess the degree of jaundice and hepatobiliary damage. Hyperbilirubinemia is considered to be a potential high-risk factor associated with postoperative mortality in hilar cholangiocarcinoma (29). A recent retrospective, single-center study once again confirmed that preoperative bilirubin concentrations were an important risk factor for postoperative severe complications and mortality in perihilar cholangiocarcinoma, with optimal cutoff values of 219.23 µmol/L and 548.08 µmol/L, respectively (30). A multicenter European study also showed that a high preoperative TBIL (≥265.2 µmol/L) was significantly associated with increased complications after the major resection of perihilar cholangiocarcinoma (31). Jaundice is associated with immune dysfunction, increased bacterial translocation, and deterioration of nutritional status and liver function (32).

Normally, bilirubin levels need to be less than twice the upper limit of normal before surgery, and preoperative biliary drainage is recommended for patients requiring decompression (TBIL > 250 µmol/L); otherwise, preoperative biliary drainage should be avoided (33). We do not have level 1 evidence for individuals with high serum bilirubin levels. Based on this study, preoperative jaundice reduction is recommended for patients with a TBIL > 194.1 µmol/L to improve performance status and survival, and preoperative biliary drainage is recommended for patients with high bilirubin (TBIL > 250 µmol/L) who require decompression.

Preoperative nutritional treatment is increasingly recognized as an important part of surgical care. Nearly 50% of hospitalized patients are malnourished or at risk of malnutrition, and hospitalized and surgical patients who are malnourished have significantly worse clinical outcomes (34). Screening for malnutrition before major surgery is essential because it can identify patients at risk of malnutrition who may benefit from preoperative nutritional intervention (35), which can improve patient outcomes (36). This study shows that preoperative PNI and TBIL levels were independent risk factors for short-term postoperative complications of dCCA. Based on the CART analysis, a high complication rate (90.24%) was found in patients with PNI ≤ 46.9 and TBIL > 194.1 µmol/L, which was useful for us to distinguish patients at potentially high risk preoperatively. Therefore, preoperative evaluations of patient nutritional status and TBIL level to help guide clinicians in formulating corresponding diagnoses and treatment strategies are expected to reduce the incidence of postoperative complications, improve quality of life, and improve the long-term prognosis of patients with dCCA.

This study has some limitations. Since the study was a retrospective analysis of data from a single center, these results may be affected by selection bias, and no further follow-up visit, more prospective, large-sample, multicenter studies are needed for further validation.

## Conclusions

Nutritional assessments are necessary before radical pancreaticoduodenectomy for dCCA. Preoperative PNI and TBIL levels can be used as predictive markers for the risk of postoperative complications. Effective perioperative intervention for patients with low PNI and high TBIL levels can further improve the surgical outcomes of patients with dCCA.

## Data Availability

The raw data supporting the conclusions of this article will be made available by the authors, without undue reservation.
