# Supplementary material for: Preoperative prognostic nutritional index predicts short-term complications after radical resection of distal cholangiocarcinoma
Source: Front Surg. 2023 Jan 10;9:1091534. doi: 10.3389/fsurg.2022.1091534 (PMC9872124; doi:10.3389/fsurg.2022.1091534)
Supplement: Supplementary file 1 [file Datasheet1.docx]

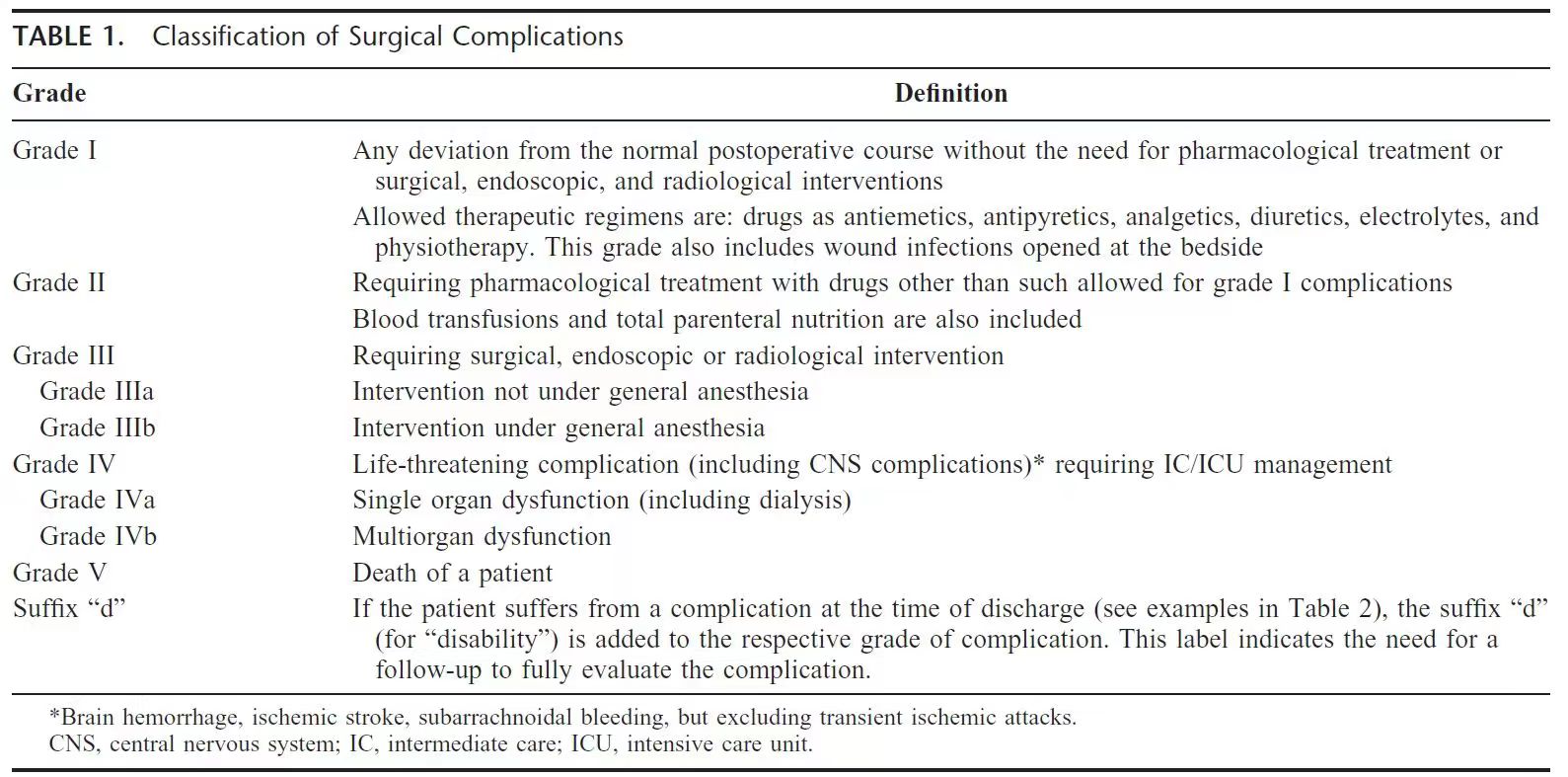


Reference: Dindo D, Demartines N, Clavien PA. Classification of surgical complications: a new proposal with evaluation in a cohort of 6336 patients and results of a survey. Ann Surg. 2004 Aug;240(2):205-13. doi: 10.1097/01.sla.0000133083.54934.ae.
